# Assessing the use of surveillance data to estimate the impact of prevention interventions on HIV incidence in cluster-randomized controlled trials

**DOI:** 10.1016/j.epidem.2020.100423

**Published:** 2020-11-20

**Authors:** Kate M. Mitchell, Dobromir Dimitrov, James P. Hughes, Mia Moore, Eric Vittinghoff, Albert Liu, Myron S. Cohen, Chris Beyrer, Deborah Donnell, Marie-Claude Boily

**Affiliations:** a Medical Research Council Centre for Global Infectious Disease Analysis, Department of Infectious Disease Epidemiology, Imperial College London, London, United Kingdom; b HIV Prevention Trials Network Modelling Centre, Imperial College London, London, United Kingdom; c Vaccine and Infectious Disease Division, Fred Hutchinson Cancer Research Center, Seattle, USA; d Department of Biostatistics, University of Washington, Seattle, USA; e Department of Epidemiology and Biostatistics, University of California San Francisco, San Francisco, USA; f Bridge HIV, Population Health Division, San Francisco Department of Public Health, San Francisco, USA; g Institute for Global Health and Infectious Diseases, University of North Carolina at Chapel Hill, Chapel Hill, NC, USA; h Department of Epidemiology, Johns Hopkins Bloomberg School of Public Health, Baltimore, USA

**Keywords:** Surveillance data, Trials, Incidence, Marker, Mathematical modelling, HIV

## Abstract

**Background:**

In cluster-randomized controlled trials (C-RCTs) of HIV prevention strategies, HIV incidence is expensive to measure directly. Surveillance data on HIV diagnoses or viral suppression could provide cheaper incidence estimates. We used mathematical modelling to evaluate whether these measures can replace HIV incidence measurement in C-RCTs.

**Methods:**

We used a US HIV transmission model to simulate C-RCTs of expanded antiretroviral therapy(ART), pre-exposure prophylaxis(PrEP) and HIV testing, together or alone. We tested whether modelled reductions in total new HIV diagnoses, diagnoses with acute infection, diagnoses with early infection(CD4 > 500 cells/μl), diagnoses adjusted for testing volume, or the proportion virally non-suppressed, reflected HIV incidence reductions.

**Results:**

Over a two-year trial expanding PrEP alone, modelled reductions in total diagnoses underestimated incidence reductions by a median six percentage points(pp), with acceptable variability(95 % credible interval −14,−2pp). For trials expanding HIV testing alone or alongside ART + PrEP, greater, highly variable bias was seen [−20pp(−128,−1) and −30pp(−134,−16), respectively]. Acceptable levels of bias were only seen over longer trial durations when levels of awareness of HIV-positive status were already high. Expanding ART alone, only acute and early diagnoses reductions reflected incidence reduction well, with some bias[−3pp(−6,−1) and −8pp(−16,−3), respectively]. Early and adjusted diagnoses also reliably reflected incidence when scaling up PrEP alone[bias −5pp(−11,1) and 10pp(3,18), respectively]. For trials expanding testing (alone or with ART + PrEP), bias for all measures explored was too variable for them to replace direct incidence measures, unless using diagnoses when HIV status awareness was already high.

**Conclusions:**

Surveillance measures based on HIV diagnoses may sometimes be adequate surrogates for HIV incidence reduction in C-RCTs expanding ART or PrEP only, if adjusted for bias. However, all surveillance measures explored failed to approximate HIV incidence reductions for C-RCTs expanding HIV testing, unless levels of awareness of HIV-positive status were already high.

## 1. Introduction

Cluster-randomized controlled trials (C-RCTs) are increasingly being used to evaluate the population-level impact of interventions on the incidence of infectious diseases, including HIV, since C-RCTs can measure the combined impact upon primary and secondary transmissions (Hayes et al., 2014; Essex, 2016; Baird et al., 2012). C-RCTs involve randomizing clusters in different arms – e.g. clinics or counties – to receive the intervention or standard of care (Donner, 1998). In each arm, incidence is measured in whole clusters or representative cohorts. C-RCTs tend to require many participants and are therefore expensive to implement and evaluate. This expense limits trial duration, typically to 2–3 years.

Costs can be reduced if routinely collected data are used to estimate trial impact on relevant outcomes. Routinely collected data have been used to measure outcomes in RCTs for non-infectious diseases, particularly in the United States (US) (Mc Cord et al., 2018, 2019; Williams et al., 2003). The HPTN 065 trial (Test, Link-to-Care Plus Treat) (Donnell et al., 2012) successfully used US surveillance data to measure levels of linkage to and continuity in HIV care and viral suppression (El-Sadr et al., 2017). Using surveillance data to estimate HIV incidence reductions in C-RCTs would be substantially cheaper than recruiting HIV incidence cohorts, and would avoid the need to conduct HIV testing in the incidence cohorts, which may in itself form an intervention in those cohorts that affects the assessed impact between intervention and control arms (Hayes et al., 2019).

While not routinely used to evaluate impact on HIV incidence in HIV prevention trials, surveillance data have been used to monitor the success of HIV prevention programs aimed at reducing HIV incidence, including the use of trends in total diagnoses to estimate the impact of expanded pre-exposure prophylaxis (PrEP) in Australia (Grulich et al., 2018) and of programs including testing, anti-retroviral therapy (ART) and PrEP in the US (CUNY Institute for Implementation Science in Population Health, 2020; Buchbinder and Havlir, 2019). The previous US National HIV/AIDS strategy had a target for reductions in new HIV diagnoses (White House Office of National AIDS Policy, 2015). Other surveillance measures used to estimate program impact include reductions in recent HIV infections (Grulich et al., 2018) and in diagnoses per test conducted (Beauchemin et al., 2018). The US Centers for Disease Control and Prevention routinely estimate national and state-level HIV incidence using surveillance data on new diagnoses and CD4 count (this approach does not have adequate precision to be used at the county level) (Song et al., 2017).

Mathematical modelling is a well-established tool for understanding the HIV epidemic in different locations (Mishra et al., 2012; Punyacharoensin et al., 2011; Stover et al., 2017) and for estimating intervention impact (Eaton et al., 2014; Cambiano et al., 2016). It has also been used to inform clinical trial design (Boily et al., 2012; Cori et al., 2014).

We used a calibrated mathematical model of HIV transmission and treatment among men who have sex with men (MSM) in the US (Mitchell et al., 2019) to inform the design of a proposed C-RCT of combination HIV prevention in this population, including provision of and support for increased HIV testing, immediate ART for HIV-infected individuals, and PrEP for at-risk uninfected individuals. During proposal development, it was suggested that county-level surveillance data on new HIV diagnoses be used to estimate cumulative HIV incidence reductions over a two-year period. We used our model to investigate if and when surveillance data on new HIV diagnoses could be used to estimate the impact of the intervention on HIV incidence in C-RCTs, and whether other routine surveillance data (e.g. new diagnoses with laboratory evidence of recent infection, levels of viral non-suppression) reflect HIV incidence reductions better than total HIV diagnoses, over different time periods and for different interventions.

## 2. Methods

### 2.1. Model

We used a previously published HIV transmission and treatment model for US MSM (Mitchell et al., 2019), and included PrEP. Briefly, it is a deterministic compartmental model of sexual HIV transmission, which divides the population by age, race, HIV infection stage and HIV care continuum engagement. Diagnosis occurs in the model when an undiagnosed HIV-infected individual tests for HIV (via routine testing or seeking treatment for symptoms). Diagnosis enables modelled HIV-infected individuals to enter HIV care and begin ART. Those on ART can achieve viral suppression, which increases their survival and greatly reduces their infectiousness to sexual partners. HIV transmission occurs through main, casual and commercial sexual partnerships, as a function of numbers of partners, frequency of sex acts, circumcision, condom and PrEP use and partners’ HIV status, infection stage, and viral suppression. Full model description, schematics (Figs. S1–S3) and equations are given in the supplementary material.

### 2.2. Model calibration

The model was previously fitted to Baltimore MSM data in a Bayesian framework involving varying 117 parameters and giving 169 parameter combinations (model fits) which reproduce well HIV prevalence trends, demography and HIV care continuum outcomes (Mitchell et al., 2019). We retained these 169 parameter combinations, and for each combination additionally selected PrEP parameter values giving PrEP coverage consistent with Baltimore MSM data (Hoots et al., 2016; Centers for Disease Control and Prevention, 2019) (details in supplementary material). Data informing model parameters and fitting ranges included National HIV Behavioural Surveillance data for Baltimore MSM, Maryland Department of Health surveillance data for Baltimore, and published literature. Model parameters and fitting data are given in Tables S1 & S2, model fits are shown in Fig. S4.

### 2.3. Model analysis

#### 2.3.1. Control (standard of care) arm

The trial control arm was simulated by running the model with each of the 169 model fits in turn from 1984 to 2024, assuming all parameters – including those governing HIV testing, treatment and PrEP initiation, adherence and retention – remained at their 2015 values until 2024.

#### 2.3.2. Intervention arm

The different trial intervention arms were simulated by running the model from 1984 to 2024, with expanded HIV testing, ART and/or PrEP from 2020 to 2024. We used Latin hypercube sampling to select 1014 intervention scenarios corresponding to different combinations of testing, linkage to care, ART and PrEP initiation, adherence and retention varied between current and plausible maximum levels. These intervention scenarios were distributed at random between the 169 original model fits; each model fit was run with six different intervention scenarios.

The intervention arm simulations, using each of the 1014 intervention scenarios, were repeated with increased levels of each intervention – HIV testing, ART or PrEP – separately (by setting increases to the other interventions to 0), and all three interventions together (using the original 1014 intervention scenarios).

In simulations for the main analysis, interventions were scaled up in all population groups equally, with a six-month ‘scale-up’ period over which HIV testing, linkage to care and PrEP initiation rates increased linearly, while improvements to other cascade parameters (ART initiation and ART and PrEP adherence and retention) occurred instantaneously. These assumptions were varied in simulations for subsequent analyses.

#### 2.3.3. Impact outcomes

In the simulated trials, true intervention impact was measured as the relative difference in the cumulative number of infections in the intervention vs. control arm. Intervention arm simulations were compared with their matched control arm simulation, ensuring identical pre-intervention incidence across arms. Impact estimates from diagnosis data were similarly measured as the relative difference in cumulative diagnoses in the intervention vs. control arm. Changes in viral non-suppression were measured as the relative difference in the proportion of diagnosed MSM not virally suppressed in the intervention vs. control arm at the end of the trial (the proportion *not* suppressed should scale proportionally with incidence reductions).

#### 2.3.4. Analyses

We compared the relative reductions in cumulative new diagnoses and cumulative new infections over two-year simulated trials expanding HIV testing, treatment and PrEP separately or simultaneously.

We simulated different trial durations – three or four years - to study whether longer trial duration influenced the comparison between reductions in new diagnoses and incidence. We compared reductions over the full trial, from the second year onwards, or over the final year of the trial, to test whether reductions in new diagnoses in later trial years had different correspondence with incidence reductions.

We studied the utility of other measures that could be estimated from routine surveillance data for estimating reductions in new infections: cumulative diagnoses with acute stage infection detected by recency assay (“acute diagnoses”) (Hall et al., 2017); cumulative diagnoses with high CD4 count (CD4 > 500 cells/μl) (“early diagnoses”); cumulative diagnoses adjusted for (divided by) total HIV tests performed (“adjusted diagnoses”) (Beauchemin et al., 2018); final proportion of diagnosed MSM virally un-suppressed.

We tested whether our results were sensitive to interventions being prioritised to higher-risk groups, to greater uptake of expanded testing provision among higher- or lower-risk groups, or to different intervention scaleup durations (one or 12 months). For completeness, we also looked at (unlikely) scenarios where testing decreased in the intervention arm.

### 2.4. Measuring how well surveillance measures reflect incidence reduction

We assessed the bias, variability and sensitivity of reductions in each surveillance marker to measure incidence reductions under different intervention scenarios.

Bias and variability statistics are adapted from Bland-Altman statistics (Bland and Altman, 1986). The bias of each surveillance measure was calculated as the median absolute difference between % reduction in that measure and % reduction in infections (% reduction in measure – % reduction in infections; expressed in percentage points, pp) across intervention runs – this indicates systematic bias which could be adjusted for by subtracting the bias from the results. Where differences varied with incidence reduction, we also assessed relative bias ((% reduction in measure – % reduction in infections)/ % reduction in infections).

We characterised the variability in each surveillance measure as the 95 % credible interval (CrI) of the absolute difference between reduction in the measure and reduction in infections (% reduction in measure – % reduction in infections; expressed in pp) across intervention runs; this measures variability over and above systematic bias, which would be difficult to adjust for and needs to be minimised. A priori we determined acceptable bounds for the 95 % CrI of ±10pp, which would result in only a modest (~16 %) increase in uncertainty (equivalently, increase in required sample size or decrease in power) for a typical HIV prevention C-RCT designed to detect a 35–40 % incidence reduction (Hayes et al., 2019; Havlir et al., 2019; Iwuji et al., 2017; Makhema et al., 2019).

We measured sensitivity to detect a true incidence reduction (which occurred in all simulated intervention scenarios) as the proportion of intervention runs where the surveillance measure was reduced (by any amount), suggesting incidence reduction. As sensitivity differed with incidence reduction, this was reported separately for ≤20 % and >20 % incidence reduction. We determined that an acceptable marker should have 100 % sensitivity to detect (clinically relevant) incidence reductions >20 % (C-RCTs of HIV prevention interventions typically aim for a 35–40 % incidence reduction (Hayes et al., 2019; Iwuji et al., 2017; Makhema et al., 2019)).

Model simulations were run in C++ and statistics calculated in R 3.6.2.

## 3. Results

### 3.1. Do reductions in HIV diagnoses reflect incidence reductions over a two-year trial?

**Fig. 1.**
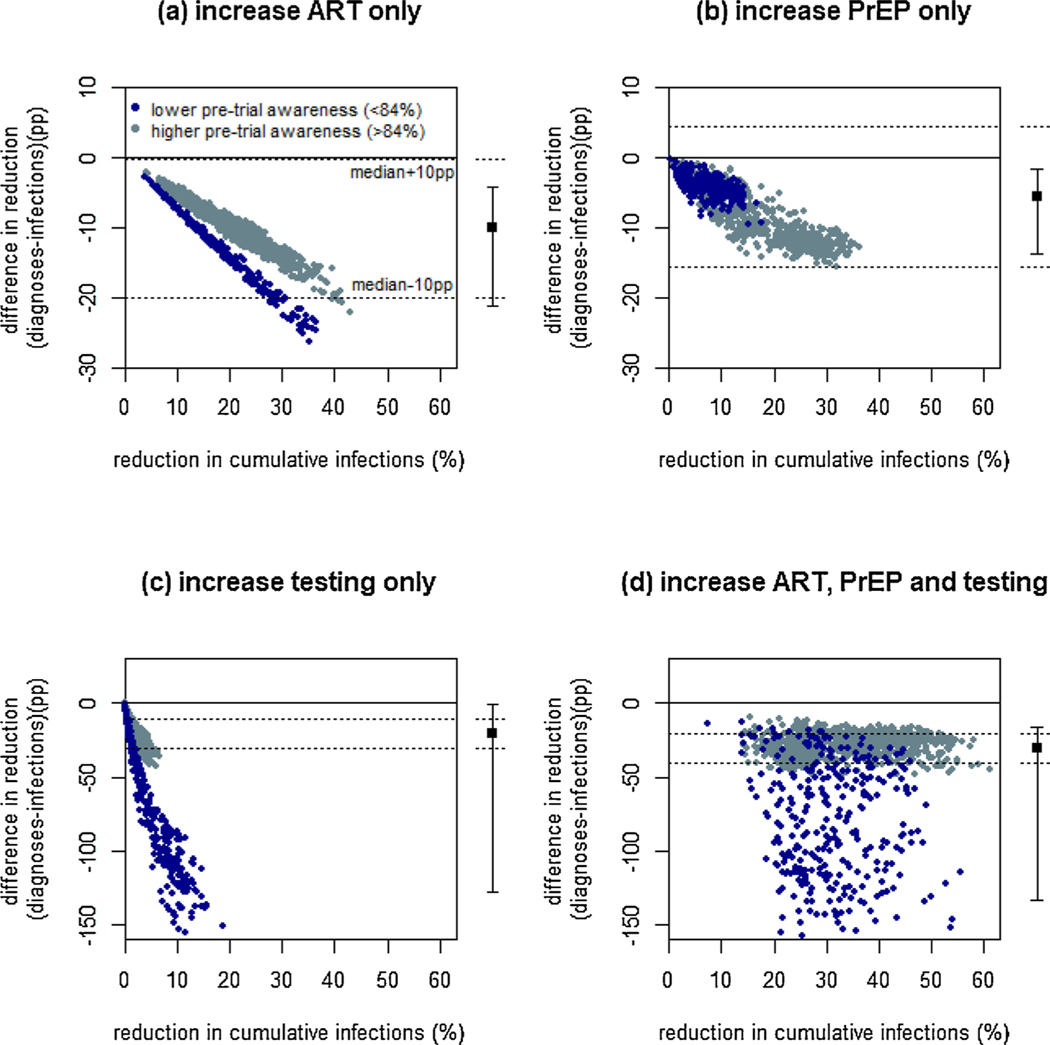
Reduction in diagnoses as a marker of reduction in infections. Shown for a two-year trial expanding (a) ART only, (b) PrEP only, (c) HIV testing only or (d) HIV testing, ART and PrEP. A six-month scale-up period is assumed. Results are shown for fits which had lower levels of pre-trial awareness of HIV-positive status (dark blue) and higher pre-trial awareness of HIV-positive status levels (grey). Each panel shows 1014 points (six different unique intervention scenarios for each of the 169 original fits). Solid line at y = 0 shows equivalence of reductions in infections and diagnoses. Dotted horizontal lines are at the median ±10pp across all points. Black filled square and error bars show median and 95 % credible interval across all points. Note the different y-axis scales used in (c) and (d).

Over a two-year trial, modelled cumulative reductions in new diagnoses always underestimated reductions in new infections caused by expansion of ART, PrEP or HIV testing, or all three simultaneously, by a median 10, 6, 20 and 30pp across all fits, respectively (Fig. 1, Table S3). This negative absolute bias increased with greater incidence reductions (Fig. 1) whereas relative bias remained constant (for interventions expanding ART alone) or decreased (for other interventions) with increasing incidence reduction (Fig. S5).

Considerable variability was seen in how well HIV diagnoses reductions reflected incidence reductions for interventions expanding HIV testing alone or in combination with other interventions, with the 95 % CrI greatly exceeding ±10pp (Fig. 1c,d, Table S3). For interventions expanding PrEP alone, the difference between diagnoses and incidence reductions always lay within ±10pp of the median (Fig. 1b), meaning HIV diagnoses could be a sufficient marker of incidence reduction in trials expanding PrEP alone, if systematic bias is adjusted for.

When HIV testing alone was expanded, the model always predicted increases in diagnoses in the intervention vs. control arm despite actual HIV incidence reductions (sensitivity 0 %; Fig. S6c). For combination interventions expanding HIV testing alongside ART and PrEP, HIV diagnoses often increased despite substantial incidence reductions (sensitivity 52 %; Fig. S6d, Table S3). All interventions expanding ART alone reduced diagnoses (sensitivity 100 %; Fig. S6a). However, diagnoses sometimes increased for PrEP interventions with small incidence reductions (sensitivity 84 % for incidence reduction <20 %), but sensitivity was 100 % if incidence declines exceeded 20 % (Fig. S6b, Table S3).

Larger biases and lower sensitivity were seen for scenarios with lower compared to higher pre-trial levels of HIV-status awareness (Fig. 1, Fig. S6, Table S3).

### 3.2. Do reductions in diagnoses better reflect incidence reductions over a longer trial, or in later trial stages?

**Fig. 2.**
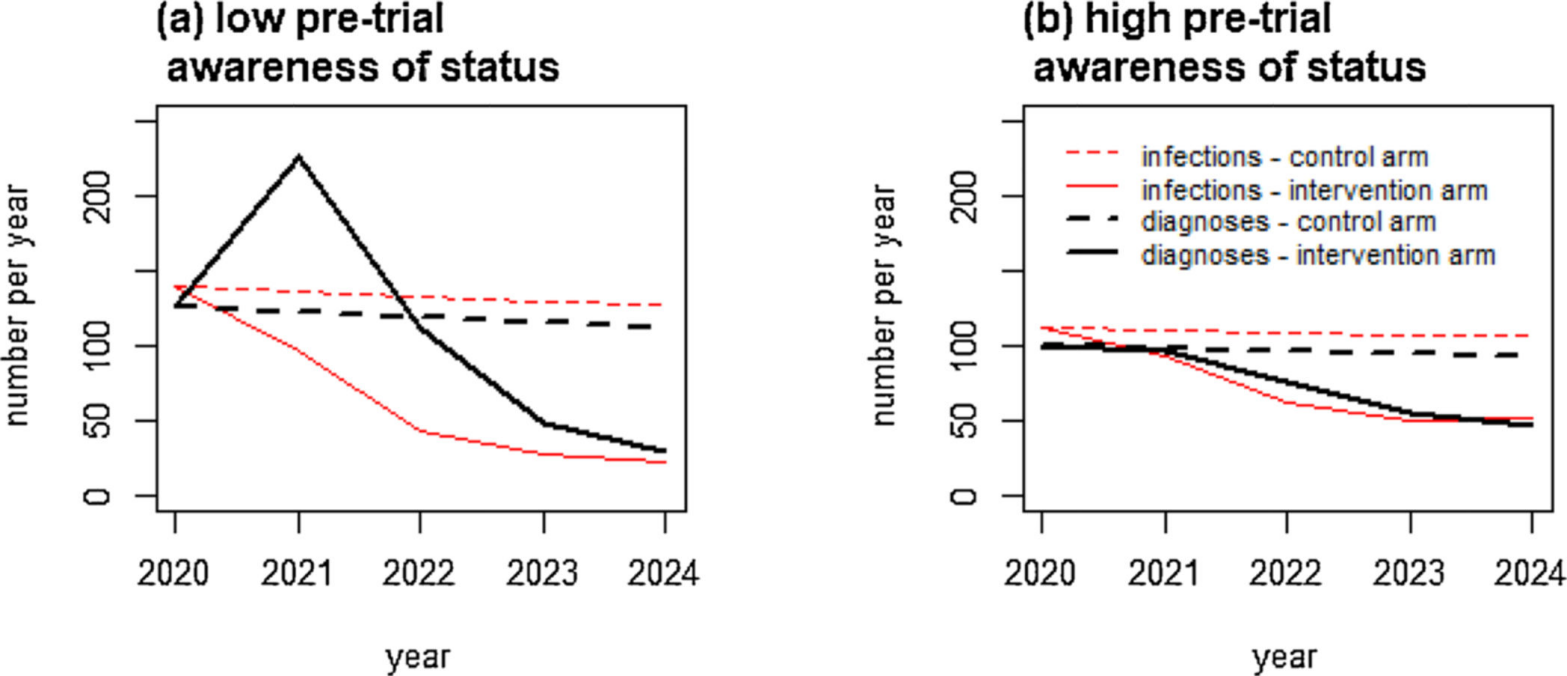
Time trends in numbers of new infections and diagnoses. Annual numbers of new infections (red lines) and diagnoses (black, thick lines) in the control arm (dashed lines) and intervention arm (solid lines), for two example parameter combinations with (a) a low level of pre-trial awareness of HIV-positive status and (b) a high level of pre-trial awareness of status, with an intervention increasing HIV testing, ART and PrEP.

When pre-trial status-awareness levels were low, interventions increasing testing caused an initial spike in new diagnoses (Fig. 2a, Fig. S7c), so diagnoses greatly overestimated new infections in the intervention arm early in the trial. Over time, the gap between intervention arm annual diagnoses and infections narrowed. With higher pre-trial awareness, a spike in diagnoses was not always seen in the intervention arm but declines in diagnoses lagged behind incidence declines, because of initial diagnoses among those already infected (Fig. 2b).

**Fig. 3.**
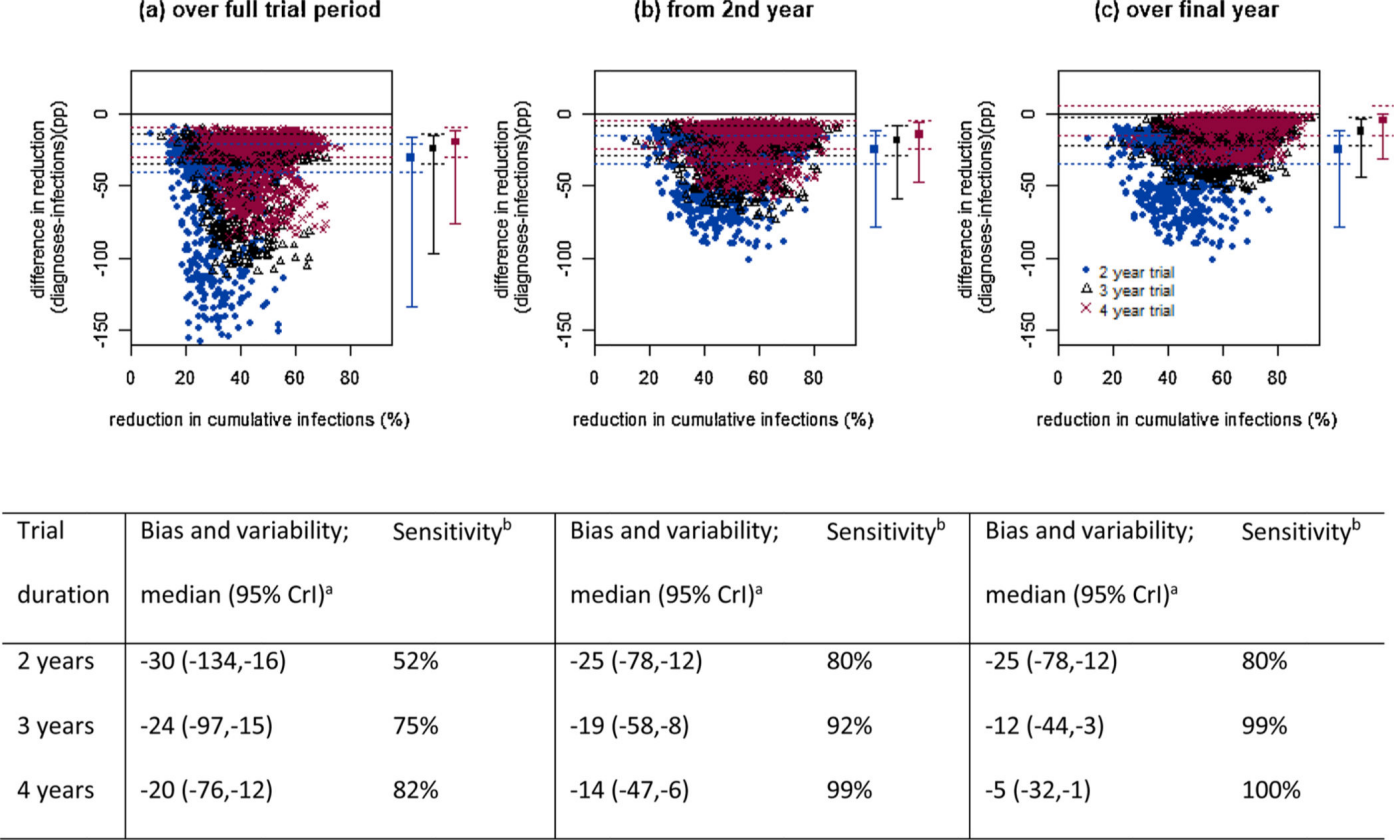
Reductions in diagnoses as a marker of reductions in infections over different time periods. Shown for a trial expanding ART + PrEP + testing, running for 2 years (blue circles), 3 years (black triangles) or 4 years (red crosses), with reductions in infections and diagnoses measured over (a) the full trial period, (b) from the second year onwards or (c) over the final year of the trial. A six-month scale-up period is assumed. Solid line at y = 0 shows equivalence of reductions in infections and diagnoses. Dotted horizontal lines are at the median ±10pp for each trial duration. Squares and error bars show median and 95 % credible interval across points for each trial duration: 2 years (blue), 3 years (black), 4 years (red). ^a^Absolute difference between reduction in diagnoses and reduction in infections (percentage points); ^b^proportion of intervention scenarios explored where reduction in diagnoses positive.

For interventions increasing testing (either alone or in combination), increasing trial duration to three or four years reduced the bias of reductions in diagnoses for estimating incidence reductions, although substantial variability remained, with the lower 95 %CrI far exceeding 10pp, and sub-optimal sensitivity (<100 %; Fig. 3a, Fig. S8g, Table S4). Measuring trial impact from the second year onward or only over the final year each further reduced bias and improved sensitivity but in all cases the lower 95 %CrI exceeded 10pp, meaning diagnoses reductions were too variable to reliably reflect incidence reductions (Fig. 3b,c, Fig. S8h,i, Table S4). For scenarios with higher pre-trial levels of awareness of HIV-positive status (≥84 %), measuring trial impact using reductions in diagnoses over a four-year trial, or from the second year or over the final year of a three- or four-year trial, reduced variability to acceptable levels (within ±10pp), and increased sensitivity to 100 %, with some bias remaining (Fig. S9a-c). With lower pre-trial awareness, diagnoses reductions were more biased, more variable, and less sensitive, and therefore not a reliable marker of incidence reductions (Fig. S9d-f). For interventions expanding ART or PrEP alone, measuring the trial impact over a longer time period or in later trial stages made little difference to the bias, variability or sensitivity of diagnoses reductions (Fig. S8a-f, Table S4).

### 3.3. Can other routine surveillance data provide better markers of incidence reduction?

**Fig. 4.**
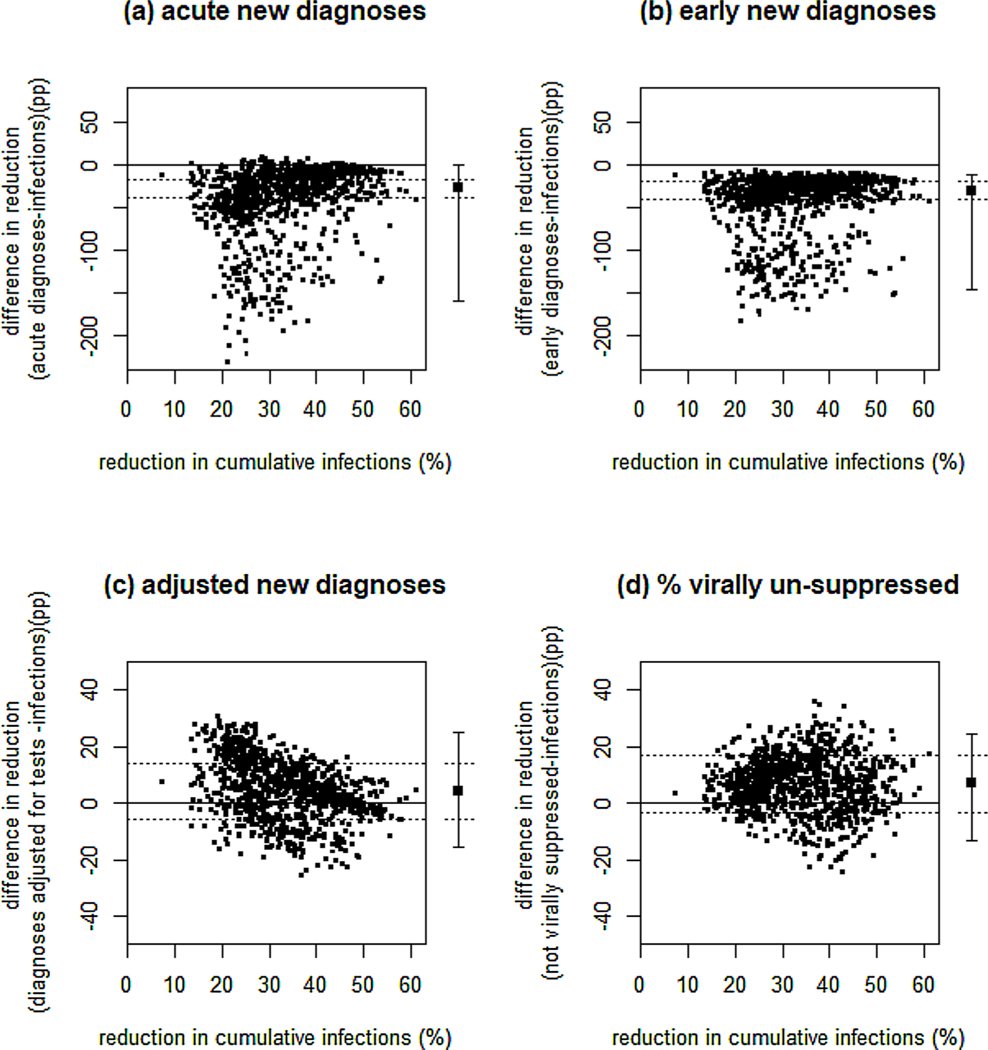
Other routine surveillance measures as markers of reduction in infections. Shown for a two-year trial expanding ART + PrEP + testing. Measures: (a) cumulative diagnoses in the acute stage of infection, (b) cumulative diagnoses with early infection (CD4 > 500 cells/μl), (c) cumulative diagnoses adjusted for the number of HIV tests performed and (d) final proportion of MSM diagnosed with HIV who are not virally suppressed. A six-month scale-up period is assumed. Solid line at y = 0 shows equivalence of reductions in infections and diagnoses. Dotted horizontal lines are at the median ±10pp across all points. Black filled square and error bars show median and 95 % credible interval across all points. Note the different y-axis scale used in (c,d).

For interventions expanding ART, PrEP and testing together, reductions in acute and early diagnoses showed comparable bias to reductions in total diagnoses for estimating incidence reductions, with similarly high variability and poor sensitivity (acute: bias −28pp, variability −159 to −1pp, sensitivity 55 %; early: bias −31pp, variability −146 to −12pp, sensitivity 53 %; Fig. 4a,b, Table S5). Reductions in early diagnoses always underestimated incidence reductions, reductions in acute diagnoses occasionally overestimated them (Fig. 4a). Reductions in adjusted diagnoses and viral non-suppression reliably identified true intervention effects (sensitivity 100 %), and had smaller bias than total diagnoses, sometimes overestimating and sometimes underestimating reductions (median difference +4pp and +7pp, respectively). However, they were still highly variable, with 95 %CrI exceeding ±10pp (Fig. 4 c, d, Table S5), rendering them poor incidence reduction markers. Even for scenarios with higher pre-trial levels of awareness of HIV-positive status (≥84 %), variability remained unacceptably high (exceeded ±10pp) for all these surveillance measures (Table S5).

For trials of interventions expanding ART, PrEP or testing alone, only the following surveillance measures were adequate markers of incidence reductions. Expanding ART alone, only reductions in acute and early diagnoses performed adequately (with 100 % sensitivity and 95 %CrI within ±10pp), with median bias −3pp and −8pp respectively (Fig. S10, Table S5). Reductions in viral non-suppression substantially overestimated incidence reductions for interventions expanding ART alone (median bias +20pp; Fig. S10d). For interventions expanding PrEP alone, in addition to total diagnoses, only reductions in early and adjusted diagnoses performed well (100 % sensitivity for incidence reductions>20 %, variability within ±10pp), with some bias (−5pp (early) and +10pp (adjusted); Fig. S11, Table S5).

### 3.4. Are relationships between reductions in surveillance markers and HIV incidence influenced by intervention prioritisation and take-up, scale-up duration, or HIV testing declines?

Compared with scenarios where interventions reached the whole population, the sensitivity of reductions in total, acute and early diagnoses and levels of viral non-suppression (measured among all MSM) were reduced when ART + PrEP + testing interventions were expanded only among higher-risk MSM (Table S6). When expanded testing was only taken up by lower-risk MSM (with PrEP and TasP expanded among all), bias was reduced and sensitivity improved for total, acute and early diagnoses, but reductions in the proportion virally unsuppressed became more biased (+21pp), compared with scenarios where all interventions reached the whole population (Fig. S13, Table S6).

Duration of intervention scale-up had little effect on the relationship between reductions in diagnoses and incidence (Table S7).

If HIV testing rates decreased in the intervention arm but remained constant in the control arm, diagnoses reductions could overestimate incidence reductions (Fig. S14).

## 4. Discussion

Using models to simulate cluster-randomized HIV prevention trials among US MSM, we found that despite their convenience, new diagnoses data captured in surveillance would generally have poor sensitivity and accuracy as a replacement measure for HIV incidence in C-RCTs and could only be used under certain conditions. The model suggested that, for interventions expanding HIV treatment only, without any change in testing rates, reductions in new diagnoses with evidence of acute HIV or early infection (CD4 >500cells/μl) reflected reductions in HIV incidence sufficiently well to envisage their use for estimating intervention impact on HIV incidence in a C-RCT. Similarly, for interventions expanding PrEP provision alone, reductions in total new diagnoses, early diagnoses or diagnoses adjusted for testing volume could potentially be used to estimate intervention impact. For each of these scenarios, adjustments of up to 10pp (made by subtracting the bias estimate from the results) would be needed to account for systematic bias. However, most interventions expanding HIV treatment or PrEP will also expand HIV testing. For interventions expanding HIV testing, alone or in combination with other interventions, reductions in total new diagnoses could only be used to estimate incidence reductions if pre-trial levels of awareness of HIV-positive status were already high (≥84 %) and over longer trial durations (at least three years). Adjustments for systematic bias (of up to 16pp) would still be required. Otherwise, none of the surveillance markers explored, including reductions in total, acute, early or adjusted diagnoses, or the proportion virally un-suppressed, reflected incidence reductions sufficiently for them to be considered as measures of impact on HIV incidence.

We found that when interventions were expanded, reductions in cumulative total HIV diagnoses always underestimated true incidence reductions, whether the intervention expanded HIV testing, treatment or pre-exposure prophylaxis, alone or in combination, even sometimes suggesting increases in HIV incidence despite true incidence declines. Particularly large bias and poor sensitivity of reductions in diagnoses to detect true incidence reductions were seen if HIV testing was expanded as part of the intervention, because increased testing tends to initially increase numbers of diagnoses as undiagnosed infected individuals are diagnosed more rapidly. Larger biases were also seen with lower levels of pre-trial awareness of HIV-positive status, as the larger pool of undiagnosed individuals means many new diagnoses are made if testing increases. For interventions expanding testing, bias was somewhat reduced and sensitivity of diagnoses reductions to detect a true incidence reduction improved over longer trial durations or when impact was measured in the later stages of the trial, as following the initial spike in diagnoses, new diagnoses subsequently reflected new infections more closely. However, even measuring diagnoses reductions over the final year of a 4-year trial expanding treatment, PrEP and testing still gave very variable estimates of incidence reductions (bias −5pp, 95 %CrI −32 to −1pp). This variability was only reduced to acceptable levels in situations with already high levels of pre-trial awareness of HIV-positive status.

While reductions in diagnoses with evidence of acute infection were a good marker of incidence reductions if ART alone were expanded, they were a poor marker if testing was expanded alone or alongside ART and PrEP. This was because increased rates of HIV testing in the intervention arm led to more people being diagnosed in the acute stage, while lower rates of testing in the control arm meant fewer individuals were diagnosed before progressing beyond acute infection. Reductions in viral non-suppression (among diagnosed individuals) underestimated the impact of expanding HIV testing or PrEP, which have other effects on HIV incidence not captured by this measure. Reductions in non-suppression overestimated the impact of expanding ART, possibly because final levels of non-suppression do not account for smaller impact during intervention scale-up.

The most relevant of our results are those with combination prevention including expansion of HIV testing as well as treatment and/or PrEP, which is most likely to be tested in a C-RCT, given the crucial role of HIV testing in accessing both treatment and PrEP. For trials including HIV testing expansion, our results suggest that surveillance measures will not be adequate markers of HIV incidence reduction, unless it is clear that levels of awareness of HIV-positive status are already high. Other methods, such as cohorts (Hayes et al., 2019) or cross-sectional incidence surveys (Coates et al., 2014), will be required to evaluate HIV incidence in C-RCTs. Note that surveillance measures may be suitable for assessing other trial goals, e.g. increasing viral suppression.

Our model was calibrated to the Baltimore HIV epidemic, and used to systematically explore the performance of surveillance measures across a wide range of epidemic and intervention scenarios, ensuring robust conclusions. While our model represents the HIV care continuum in detail it may not fully capture heterogeneity in HIV testing rates between individuals. However, we expect the discrepancy between reductions in diagnoses and new infections to persist regardless of the precise pattern of testing assumed. Our model does not account for testing errors or inaccuracies (e.g. for acute infection or viral suppression), which would be expected to increase variability in how well these surveillance measures reflect incidence reductions, reducing their suitability as markers. We have not considered contamination between trial clusters, which could reduce ability to detect a true incidence reduction but is not expected to affect agreement between surveillance markers and incidence reduction. Finally, we do not consider stochastic or sampling variation between clusters, which would reduce the precision of these surveillance measures to estimate incidence reduction, particularly for measures with fewer endpoints, such as acute diagnoses.

Using routinely-collected data may help to obtain impact results faster for RCTs of non-infectious diseases interventions (Mc Cord et al., 2019). For HIV intervention trials, however, reporting delays following HIV diagnosis mean that using surveillance data may delay trial results, and make timely interim analysis difficult. Our finding that interventions increasing HIV testing lead to surveillance diagnoses data giving very biased incidence estimates agrees with findings from non-infectious disease trials that routinely-collected data could give biased results if an intervention leads to more contact with healthcare professionals collecting routine data (Mc Cord et al., 2019).

## 5. Conclusions

Our analysis robustly demonstrated that data on new HIV diagnoses and other information collected in routine HIV surveillance (e.g. acute infection at diagnosis, viral suppression) cannot provide reliable estimates of reductions in new HIV infections in C-RCTs of HIV interventions when HIV testing is included in the intervention package, unless high levels of awareness of HIV-positive status have already been achieved in the population (by high rates of HIV testing). Reductions in some surveillance diagnoses measures may be useful surrogates for HIV incidence in C-RCTs expanding ART or PrEP only, although a bias adjustment is needed. As increased HIV testing is essential for HIV prevention in many settings, this greatly limits the use of such routine surveillance data for estimating intervention impact on HIV incidence in C-RCTs.

## Supplementary Material

1

## Abbreviations:

CrI: credible interval
pp: percentage points
